# External quality assessments for SARS-CoV-2 genome detection in Austria: A comparison of the first postpandemic round with results from the pandemic era

**DOI:** 10.1007/s00508-024-02353-1

**Published:** 2024-04-23

**Authors:** Christoph Buchta, Stephan W. Aberle, Irene Görzer, Andrea Griesmacher, Mathias M. Müller, Erich Neuwirth, Elisabeth Puchhammer-Stöckl, Lukas Weseslindtner, Jeremy V. Camp

**Affiliations:** 1 Austrian Association for Quality Assurance and Standardization of Medical and Diagnostic Tests (ÖQUASTA), Hörlgasse 18/5, 1090 Vienna, Austria; 2 Center for Virology, Medical University of Vienna, Kinderspitalgasse 15, 1090 Vienna, Austria; 3 Central Institute for Medical and Chemical Laboratory Diagnosis, Innsbruck University Hospital, Innsbruck, Austria; 4 Faculty of Computer Science, University of Vienna, Vienna, Austria

**Keywords:** COVID-19, Diagnostics, Virus genome detection, PCR, NAT, Molecular

## Abstract

**Background:**

External quality assessment (EQA) schemes provide objective feedback to participating laboratories about the performance of their analytical systems and information about overall regional analytical performance. The EQAs are particularly important during pandemics as they also assess the reliability of individual test results and show opportunities to improve test strategies. With the end of the COVID-19 pandemic, the testing frequency significantly decreased in Austria. Here, we analyzed whether this decrease had an effect on participation and/or performance in SARS-CoV‑2 virus detection EQAs, as compared to the pandemic era.

**Material and methods:**

Identical samples were sent to all participating laboratories, and the EQA provider evaluated the agreement of the reported results with defined targets. The EQA was operated under two schemes with identical samples and therefore we analyzed it as a single EQA round. The performance of testing was reported as true positive ratios, comparing the post-pandemic data to previous rounds. Furthermore, subgroups of participants were analyzed stratified by laboratory type (medical or nonmedical) and the test system format (fully automated or requiring manual steps).

**Results:**

While the frequency of false negative results per sample did not change during the 3 years of the pandemic (5.7%, 95% confidence interval [CI] 3.1–8.4%), an average per sample false negative ratio of 4.3% was observed in the first post-pandemic EQA (0%, 1.8%, and 11% for the 3 positive samples included in the test panel, *n* = 109 test results per sample). In this first post-pandemic EQA medical laboratories (average 0.4% false negative across 3 samples, *n* = 90) and automated test systems (average 1.2% false negative, *n* = 261) had lower false negative ratios than nonmedical laboratories (22.8%, *n* = 19) and manual test systems (16.7%, *n* = 22). These lower average ratios were due to a low concentration sample, where nonmedical laboratories reported 36.8% and manual test systems 54.5% true positive results.

**Conclusion:**

Overall ratios of true positive results were below the mean of all results during the pandemic but were similar to the first round of the pandemic. A lower post-pandemic true positive ratio was associated with specific laboratory types and assay formats, particularly for samples with low concentration. The EQAs will continue to monitor the laboratory performance to ensure the same quality of epidemiological data after the pandemic, even if vigilance has decreased.

**Supplementary Information:**

The online version of this article (10.1007/s00508-024-02353-1) contains supplementary material, which is available to authorized users.

## Introduction

Diagnostic testing for infectious agents is essential to identify symptomatic or asymptomatic infected individuals and is therefore a pillar in the management of epidemics, as recently experienced in the coronavirus disease 2019 (COVID-19) pandemic. The COVID-19 pandemic presented a challenging situation in which many different test systems were implemented for the first time, as they were new to the market, and their performance in routine testing use was hardly known. Similarly, the rapid expansion of testing capacity in the shortest possible time required by public health authorities meant that tests were carried out by entities whose competence was not necessarily based on pre-existing qualifications and experience with such laboratory activities, namely virus diagnostics. Whether these circumstances affected the analytical performance was an important question, as the reliability of SARS-CoV‑2 test results came under scrutiny in both public and professional fields [1].

External quality assessment (EQA) programs provide laboratories with information on the performance of their test system in routine use and in comparison with other test systems that analyze identical samples simultaneously. For manufacturers of test systems and registration authorities, results and data from EQA schemes are of essential importance for complying with the obligation to ensure post-market surveillance required by international regulations on in vitro diagnostics (IVD) [2]. Furthermore, as the results of pathogen detection tests form the basis for epidemiological indicators used by public health authorities, pathogen detection EQA data provide insights into the reliability of epidemiological monitoring [3].

In March 2020 the COVID-19 outbreak was declared a pandemic and the key message from the World Health Organization (WHO) Director-General was to increase test frequencies [4, 5]. By following this call, Austria was among the countries with the highest number of pathogen detection tests per thousand inhabitants in the world [6]. In a recent study we investigated the performance of SARS-CoV‑2 virus genome detection in Austrian EQA schemes during the 3‑year COVID-19 pandemic [7] (summarized in Table 1) and 38 months later, in May 2023, the pandemic was declared over [8]. For laboratories, not only in Austria, this dramatically changed the situation: public funding no longer covers test costs, the daily number of tests performed has plummeted, and many test facilities have stopped operations; however, as epidemiological monitoring is still important, the testing continues, as should EQA schemes. Therefore, we analyzed in this study whether the changed testing situation has affected the overall testing performance in Austrian EQAs. In particular, we report on the first post-pandemic EQA in Austria for SARS-CoV‑2 virus genome detection, as compared to the outcomes of all earlier rounds.

**Table 1:** Performance in SARS-CoV‑2 nucleic amplification testing as observed by external quality assessment during 3 years of COVID-19 pandemic compared to post-pandemic [7]

	Pandemic	Post-pandemic
**True positive results**	**4431/4723 (93.8%)**	**312/327 (95.4%)**
*Performance of assay types*		
Automated	721/732 (98.5%)	42/42 (100%)
Automated NPT/POCT^a^	1818/1916 (94.9%)	215/219 (98.2%)
Manual	1892/2075 (91.2%)	55/66 (83.3%)
*Performance of laboratory types*		
Medical	3002/3128 (96.0%)	268/270 (99.3%)
Nonmedical	1198/1338 (89.5%)	35/42 (83.3%)
Pharmacies	231/257 (89.9%)	9/15 (60.0%)
**False negative results**	**255/4738 (5.4%)**	**14/327 (4.3%)**
*Performance of assay types*		
Automated	10/732 (1.4%)	0/42 (0.0%)
Automated NPT/POCT	67/1916 (3.5%)	3/219 (1.4%)
Manual	164/2075 (7.9%)	11/66 (16.7%)
*Performance of laboratory types*		
Medical	106/3128 (3.4%)	1/270 (0.4%)
Nonmedical	110/1338 (8.2%)	7/42 (16.7%)
Pharmacies	25/257 (9.7%)	6/15 (40.0%)
**Inconclusive results**	**52/4738 (1.1%)**	**1/327 (0.3%)**
*Performance of assay types*		
Automated	1/732 (0.1%)	0/42 (0%)
Automated NPT/POCT	31/1916 (1.6%)	1/219 (0.5%)
Manual	19/2075 (0.9%)	0/66 (0%)
*Performance of laboratory types*		
Medical	20/3128 (0.6%)	1/270 (0.4%)
Nonmedical	30/1338 (2.2%)	0/42 (0%)
Pharmacies	1/257 (0.4%)	0/15 (0%)

^a^Near patient test/point of care test

## Material and methods

The Austrian SARS-CoV‑2 virus genome detection schemes are operated by the EQA provider, the Austrian Association for Quality Assurance and Standardization of Medical and Diagnostic Tests (ÖQUASTA), in cooperation with the national reference laboratory for respiratory viruses, the Center for Virology of the Medical University of Vienna. There were two EQA schemes for virus genome detection, one of which targeted pharmacies, as they were only allowed to use near patient test/point of care test (NPT/POCT) systems. For the post-pandemic EQA, a total of 116 and 14 participants were registered for the SARS-CoV‑2 virus genome detection and POCT EQA schemes, respectively, both conducted within August 2023. For both schemes, the same samples were used, dispatched on the same date, and therefore the combined data are presented and analyzed. The samples passed stability and homogeneity tests (multiple testing and testing after storage to mimic extreme shipping conditions, as described previously [7]) and were shipped to participants under ambient conditions. Participants were advised to store the samples for as short a time as possible at 2–8 °C before examination and to analyze them in the same way as routine clinical samples. As recommended, the test results were reported to the EQA provider within 12 days as “positive (SARS-CoV‑2 RNA detected)”, “negative (SARS-CoV‑2 RNA not detected)” or “inconclusive” and stating the test system used. A web portal, e‑mail, fax or post were available for this purpose. The EQA provider compared submitted results with the targets for the individual samples and if there was a match, the respective result was rated as “correct”, otherwise as “incorrect”. Participants received confidential individual reports. The aggregated results of the performance of all participant test systems were presented in a summary report.

### Specifications of samples

Sets containing 900 µL each of 5 different sample materials (S1–S5) were prepared for the first post-pandemic EQA in August 2023. Positive samples were either produced by diluting residual clinical specimens (S1, S4) or a standard (S2) with phosphate-buffered saline (PBS) ([9]; Table 2). Negative samples were either PBS (S3) or a clinical sample negative for SARS-CoV‑2, but positive for influenza A(H1N1) diluted with PBS (S5) (Table 2). Sample S1 also included respiratory syncytial virus RNA and, therefore, S1 and S5 served as tests of specificity, while the diluted standard (S2) served as a sensitivity test. Previously, there were 51 samples positive for SARS-CoV‑2 used in the SARS-CoV‑2 virus genome detection EQA schema performed since May 2020. On three occasions (May 2022, August 2022 and once during the post-pandemic period in August 2023), the virus genome detection EQA scheme and the POCT scheme were conducted nearly simultaneously using the same sample panel, and therefore there were 14 unique EQA rounds during the pandemic and 1 during the post-pandemic time period (i.e., a total of 17 rounds but with 14 unique sample panels). Standards (Accuplex SARS-CoV‑2 molecular controls kit; SeraCare; Millford, MA, USA) diluted to target concentrations of 1000 copies/mL (cp/mL) were present in 5 rounds as well as in the first post-pandemic rounds (total 11 samples). These allowed comparison of performance indicators over time across several EQA rounds and on a per sample basis.

**Table 2:** Specifications of samples S1–S5 used in two simultaneous EQA rounds for SARS-CoV‑2 virus genome detection in August 2023

Sample specifications	S1	S2	S3	S4	S5
SARS-CoV‑2 virus genome	Positive	Positive	Negative	Positive	Negative
Origin of sample	Clinical specimen	Diluted standard^a^	PBS	Clinical specimen	Clinical specimen
SARS-CoV‑2 virus variant	XBB	n/a	n/a	XBB	n/a
Ct value	28.1	35.8	n/a	24.7	n/a
Cp/mL	~140,000	~1000	n/a	~1,100,000	n/a
Matrix	PBS	PBS	PBS	PBS	PBS
Additional viruses in the sample	RSV‑B	–	–	–	Influenza A—H1

^a^AccuPlex SARS-CoV‑2 Reference Material Full Genome (SeraCare Life Sciences, Inc.), lot 10593976

*n/a* not applicable, *PBS* phosphate-buffered saline, *RSV* respiratory syncytial virus

### Classification of participants

Participants were classified as medical (registered medical diagnostic laboratories, hospital diagnostic laboratories or special care clinics and microbiological or virological departments within university hospitals) or nonmedical laboratories (blood banks, academic teaching and/or research laboratories, military and governmental laboratories, general practitioners and walk-in clinics, distributors/manufacturers of diagnostic tests, and laboratories dedicated solely to SARS-CoV‑2 testing). From 2022, pharmacies (which we classify as a type of nonmedical laboratory) were serviced in their own EQA scheme as they were allowed to exclusively use test systems approved for near patient test/point of care test (NPT/POCT) use (which we classify as a type of automated test system) [10].

### Classification of test systems

The test systems used were classified as automated laboratory test systems (no manual extraction or purification steps required) or manual test methods (manual extraction and/or purification steps, use of multi-well cyclers but using approved CE IVD labelled reagents). Some laboratories reported using in-house test systems as a special form of manual test methods (manual test methods using laboratory developed in-house reagents). We classified NPT/POCT test systems (test systems specifically approved for point of care use or meeting the relevant requirements) as automated systems.

### Statistics

**Fig. 1:**
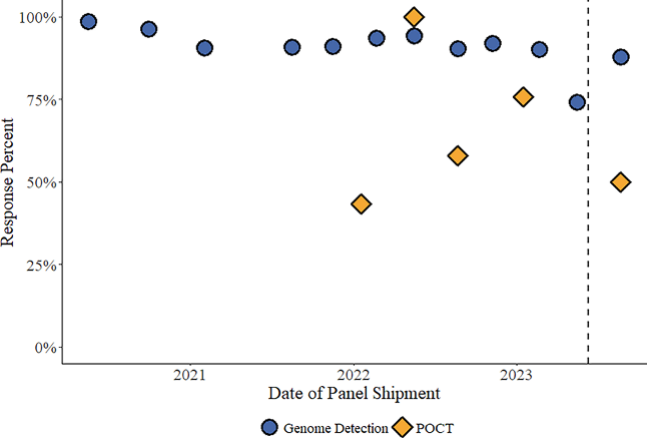
Response ratios in the SARS-CoV‑2 virus genome detection scheme (*blue circles*) and a separate EQA scheme dedicated for users of point-of-care tests (POCT; *orange diamonds*) from 2020–2023. These two schemes were conducted simultaneously three times (twice during the pandemic and once post-pandemic, vertically aligned in the figure), and consisted of the same sample panels dispatched at the same time. *Vertical dashed line* indicates the end of the pandemic

The true positive, false positive and negative ratios were calculated for the aggregated results, and these are expressed as percentages. We calculated the per sample expected sensitivity (true positive, true positive + false negative) as a function of sample concentration (based on mean reported Ct value for E gene RT-qPCR results) using all pandemic EQA rounds with a mixed effects logistic regression model, as previously described [7] and compared the post-pandemic EQA results to the 95% confidence interval. As the results were analyzed on a per sample basis, it was important to combine results from identical samples that were dispatched under the two EQA schemes. Details about 12 of the 13 unique pandemic EQA rounds have been previously published [7] and the data here include the previously unpublished data from the round performed in May 2023 (Table 1; Fig. 1). Similarly, we tested the performance over time by calculating the mean (and 95% confidence interval) for all samples with approximately 1000 copies/mL and comparing the data from the post-pandemic EQA to that, stratifying by laboratory type or assay format. As the data set was structured in a way that some potentially confounding variables could not be statistically accounted for (e.g., multiple tests submitted by some but not all laboratories, where laboratory participation occurred irregularly over time), we limited our inferences to these simple statistical comparisons.

## Results

### Participation and response ratios after and during the pandemic

In the first post-pandemic EQA (both schemes combined), 96 unique participants registered and reported results from at least 1 test system, 1 of which reported results from 5 test systems, 2 from 3, and 5 from 2 test systems for a total of 109 responses (Table 3). Most of the participants were registered in the regular scheme (91 unique participants reporting 102 responses), while 6 participants reported results from 1 test system and 1 reporting 2 test systems in the POCT scheme (one participant that reported results from one test system in the POCT scheme also participated with two test systems in the regular scheme). In the EQA rounds during the pandemic, the response ratios in the SARS-CoV‑2 virus genome detection scheme decreased from 99% to 74% (a rate of −0.3%/month, *p* = 0.018), and in the SARS-CoV‑2 POCT scheme it varied between 43% and 100% (Fig. 1). In the post-pandemic rounds 88% (102/116) of the participants reported results (for at least 1 sample) in the SARS-CoV‑2 virus genome detection scheme, and 50% (7/14) in the SARS-CoV‑2 POCT scheme (Fig. 1).

**Table 3:** Results obtained in the first post-pandemic EQA for samples positive for SARS-CoV‑2 virus genome (August 2023)

Results sample	S1	S2	S4	Total
True positive results	107/109 (98.2%)	97/109 (89.0%)	108/109 (99.1%)	312/327 (95.4%)
False negative results	2/109 (1.8%)	12/109 (11.0%)	0/109 (0.0%)	14/327 (4.3%)
Inconclusive results	0/109 (0.0%)	0/109 (0.0%)	1/109 (0.9%)	1/327 (0.3%)
**Performance of laboratory types**				
*Medical laboratories*				
True positive	89/90 (98.9%)	90/90 (100%)	89/90 (98.9%)	268/270 (99.2%)
False negative	1/90 (1.1%)	0/90 (0.0%)	0/90 (0.0%)	1/270 (0.4%)
Inconclusive	0/90 (0.0%)	0/90 (0.0%)	1/90 (1.1%)	1/270 (0.4%)
*Nonmedical laboratories*				
True positive	14/14 (100%)	7/14 (50.0%)	14/14 (100%)	35/42 (83.3%)
False negative	0/14 (0.0%)	7/14 (50.0%)	0/14 (0.0%)	7/42 (16.7%)
*Pharmacies*				
True positive	4/5 (80.0%)	0/5 (0.0%)	5/5 (100%)	9/15 (60.0%)
False negative	1/5 (20.0%)	5/5 (100%)	0/5 (0.0%)	6/15 (40.0%)
**Performance of assay types**				
*Automated laboratory systems*				
True positive results	14/14 (100%)	14/14 (100%)	14/14 (100%)	42/42 (100%)
False negative results	0/14 (0.0%)	0/14 (0.0%)	0/14 (0.0%)	0/42 (0.0%)
*Automated systems intended for NPT/POCT use*				
True positive	72/73 (98.6%)	71/73 (97.3%)	72/73 (98.6%)	215/219 (98.2%)
False negative	1/73 (1.4%)	2/73 (2.7%)	0/73 (0.0%)	3/219 (1.4%)
Inconclusive	0/73 (0.0%)	0/73 (0.0%)	1/73 (1.4%)	1/219 (0.5%)
*Manual methods*				
True positive	21/22 (95.5%)	12/22 (54.6%)	22/22 (100%)	55/66 (83.3%)
False negative	1/22 (4.6%)	10/22 (45.4%)	0/22 (0.0%)	11/66 (16.7%)

### Overall analytical sensitivity and specificity in post-pandemic rounds

**Fig. 2:**
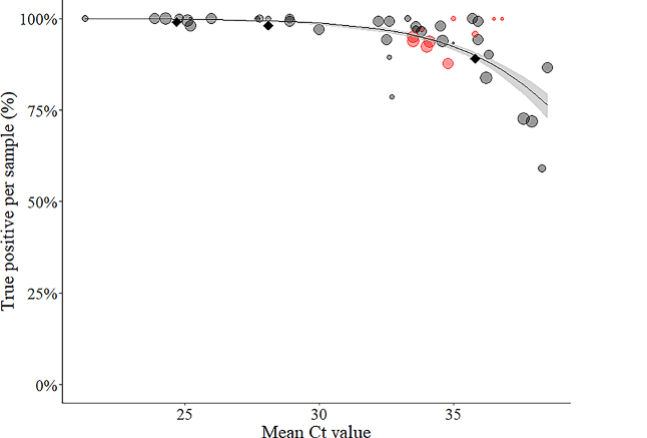
Sensitivity, as estimated by true positive percent per sample, of results submitted to SARS-CoV‑2 genome detection EQAs as a function of virus concentration (based on mean estimated Ct value of RT-qPCR assays targeting the viral E gene). *Circles* show all assays since the beginning of the pandemic, with *red circles* indicating standards diluted to target of 1000 copies/mL. *Black diamonds* show the performance for three samples included in the first post-pandemic EQA rounds (S1, S2, and S4). The sizes of the circles are relative to the number of submitted results per sample (*N* = 28–171). The line indicates the expected mean sensitivity as estimated by mixed effects logistic regression and the gray band is the 95% confidence interval around this expected value

In the post-pandemic rounds a total of 327 results were submitted for the 3 samples positive for SARS-CoV‑2 (Table 3). Among them, 95.4% (312/327) were true positive, 4.3% (14/327) were false negative, and 0.3% (1/327) were inconclusive (Table 3). Based on the EQA rounds during the pandemic, the expected true positive ratio per sample was 94.2% (91.6–96.9%), but varied according to sample concentration (Fig. 2) and the average per sample false negative ratio was 5.7% (95% CI 3.1–8.4%) [7]. The sample S1 (~140,000 cp/mL, mean Ct 28.1) was tested true positive by 98.2% and false negative by 1.8% of the participants in both schemes; S2 (~1000 cp/mL, mean Ct 35.8) was tested true positive by 89.0%, and false negative by 11.0%; S4 (~1,100,000 cp/mL, mean Ct 24.7) was tested true positive by 99.1% and inconclusive by 0.9% (Table 3). The true positive ratios for S1 and S4 were slightly less than the expected values for samples of a similar concentration (99.1–99.6% and 99.7–99.9% for Ct values of 28.1 and 24.7, respectively), but the value for S2 was within the confidence interval (88.7–90.9% for Ct value 35.8) (Fig. 2). All 218 results reported for the 2 samples in the panel negative for SARS-CoV‑2 were reported true negative (data not shown).

### Performance of different types of participants and test systems in post-pandemic rounds

A total of 90 medical laboratories reported 99.2% (268/270) true positive and each 0.4% (1/270) false negative and inconclusive results (Table 3). A total of 19 nonmedical laboratories reported 77.2% (44/57) true positive and 22.8% (13/57) false negative results, with no inconclusive results (Table 3). Among the nonmedical laboratories, 5 pharmacies reported 60% (9/15) true positive and 40% (6/15) false negative results (Table 3).

Participants in post-pandemic assessment used 36 different test systems (combinations of 23 devices and 33 reagents). Among those were 8 automated laboratory test systems, with an additional 9 classified as POCT assays, 19 manual methods, and no in-house assays (Supplement 1). For the positive samples, 98.5% (257/261) of all automated systems, including those intended for NPT/POCT use, results were true positive, with 3 (1.2%) false negative and 1 (0.3%) inconclusive results (Table 3). The majority (83.9%, 219/261) of automated test systems could be classified as NPT/POCT systems, providing 98.2% (215/219) of results as true positive, 1.4% (3/219) as false negative, and < 0.5% (1/219) as inconclusive (Table 3). A total of 83.3% (55/66) of results obtained by manual methods were true positive, and 16.7% (11/66) were false negative (Table 3).

### Results reported for samples at ~1000 cp/mL in earlier and post-pandemic rounds

Over all rounds, a total of 93.1% (965/1037) results reported for the 11 samples with ~1000 cp/mL were true positives, 6.2% (64/1037) were false negatives, and 0.8% (8/1037) were inconclusive (Table 4). The mean Ct values for the E gene for these samples was between 33.5 and 36.8 (average 34.9, SD ±1.2) (Table 2 and [7]). Medical laboratories reported 96.0% (652/679) of these samples as true positive, 3.4% (23/679) as false negative, and 0.6% (4/679) as inconclusive; nonmedical laboratories (including pharmacies) reported 87.4% (313/358) samples as true positive, 11.4% (41/358) as false negative, and 1.1% (4/358) inconclusive (Table 4). Among the nonmedical laboratories, 33 pharmacies participated at various times across 5 of the 6 EQA rounds (9–16 per round) in which sample(s) with ~1000 cp/mL were included, comprising 83 of the 358 results from nonmedical laboratories (Table 4). Pharmacies reported 79.5% (66/83) samples as true positive and 20.5% (17/83) as false negative, reporting 100% of samples negative in the first (Nov 2021, *n* = 9 pharmacies) and the 5 most recent rounds, but on average returning 95.6% (66/69) true positive results in the other 3 rounds (Table 4).

**Table 4:** True positive and false negative results as obtained by different types of participants using different types of assay types for 11 samples with a virus load of ~1000 cp/mL in seven SARS-CoV‑2 genome detection EQA events

	Medical Laboratories	Nonmedical laboratories	Pharmacies	Total
**True positive results**				
Automated laboratory systems	146/148 (98.6%)	11/11 (100%)	–	157/159 (98.7%)
Automated systems for NPT/POCT use	377/386 (97.7%)	45/57 (78.9%)	11/13 (84.6%)	433/456 (95.0%)
Manual methods	117/129 (90.7%)	189/204 (92.6%)	55/70 (78.6%)	361/403 (89.6%)
In-house assays	12/16 (75.0%)	2/3 (66.7%)	–	14/19 (73.7%)
*Total*	652/679 (96.0%)	247/275 (89.8%)	66/83 (79.5%)	965/1037 (93.1%)
**False negative results**				
Automated laboratory systems	2/148 (1.4%)	0/11 (0.0%)	–	2/159 (1.3%)
Automated systems for NPT/POCT use	7/386 (1.8%)	8/57 (14.0%)	2/13 (15.4%)	17/456 (3.7%)
Manual methods	10/129 (7.8%)	15/204 (7.4%)	15/70 (21.4%)	40/403 (9.9%)
In-house assays	4/16 (25.0%)	1/3 (33.3%)	–	5/19 (26.3%)
*Total*	23/679 (3.4%)	24/275 (8.7%)	17/83 (20.5%)	64/1037 (6.2%)
**Inconclusive results**				
Automated laboratory systems	0/148 (0.0%)	0/11 (0.0%)	–	0/159 (0.0%)
Automated systems for NPT/POCT use	2/386 (0.5%)	4/57 (7.0%)	0/13 (0.0%)	6/456 (1.3%)
Manual methods	2/129 (1.6%)	0/204 (0.0%)	0/70 (0.0%)	2/403 (0.5%)
In-house assays	0/16 (0.0%)	0/3 (0.0%)	–	0/19 (0.0%)
*Total*	4/679 (0.6%)	4/275 (1.5%)	0/83 (0.0%)	8/1037 (0.8%)

**Fig. 3:**
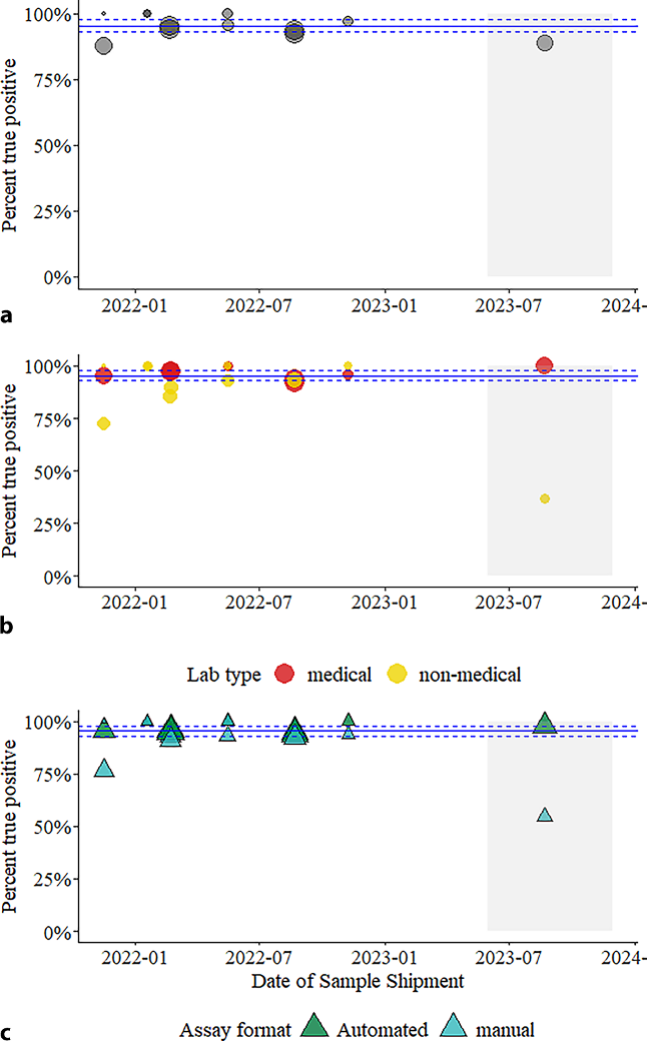
Percent true positive per sample for SARS-CoV‑2 virus genome detection results submitted to EQAs for samples with approximately 1000 copies/mL. **a** Each point represents the percent true positive results submitted for seven EQA events, including one post-pandemic sample in gray background, with panels that contained one or more standard samples with a target dilution of 1000 copies/mL. The solid horizontal blue line is the mean of all results submitted during the pandemic, with the horizontal dashed lines the 95% confidence interval, for samples of the same concentration. The middle and bottom panels show the same data stratified by **b** laboratory type (medical as *red circles* or nonmedical as *yellow circles*) and **c** assay format (automated including POCT/NPT assay as *green triangles* or assays requiring at least one manual step as *blue triangles*). The *grey* boxes show the post-pandemic period. The size of the circles or triangles is relative to the number of results for that sample (*N* = 28–171)

A total of 98.7% (157/159) of results reported by automated laboratory test systems were true positive, 1.3% (2/159) were false negative, and 0.0% were inconclusive and, in addition to those, automated test systems intended for NPT/POCT use reported 95% (433/456) true positive, 3.7% (17/456) false negative, and 1.3% (6/456) inconclusive results (Table 4). Manual test systems reported 89.6% (361/403) true positive, 9.9% (40/403) false negative, and 0.5% (2/403) inconclusive results and laboratory developed (in-house) test systems reported 73.7% (14/19) true positive, 26.3% (5/19) false negative, and no inconclusive results (Table 4). The percent true positives in the post-pandemic round (89.0%) for the low-concentration sample was below the 95% CI (92.4–97.4%) based on samples of similar concentration from previous rounds (Fig. 3a). Notably, medical laboratories (100% true positive) and automated test systems (97.7% true positive) had true positive ratios higher than this interval for low-concentration sample in the post-pandemic round (Fig. 3b and c). Conversely, nonmedical laboratories and automated laboratory systems had much lower proportions of true positive results (Table 4), significantly outside the expected value for low concentration samples based on the 95% C.I. of previous rounds (Fig. 3b and c).

## Discussion

In this study, we report the results from the first post-pandemic EQA for SARS-CoV‑2 virus genome detection and compare these results to the previous rounds. The aim was to determine whether the overall performance had changed since the pandemic ended, given that specific testing circumstances have changed. As a main finding we show that the response ratio of registered laboratories for the genome detection EQA schemes continuously dropped as the pandemic progressed, from 99% to 74% at a rate of −0.3% per month (Fig. 1). This decrease may be related to a loss of interest in prioritizing SARS-CoV‑2 genome detection assays, or the impression that assays have been sufficiently validated. As there are no data on the number of test facilities that were in operation in Austria at a specific time and which test systems were used, no statement can be made as to what proportion complied with the statutory obligation to participate in EQA. The only available information in this respect is the number of 1034 pharmacies registered to carry out tests in Austria in January 2023. We note that the national SARS-CoV‑2 POCT EQA scheme at this time had only 28 participants [16], and we report variable participation in the POCT EQA scheme over time (Fig. 1). The emergence of novel genetic and antigenic variants provides an impetus for laboratories to continue monitoring genome detection assays through EQA; however, ultimately, we do not know the precise individual motivation(s) that drove participation in EQAs and, more importantly, the reasons for not reporting results when a participant has registered for a given round.

The overall performance in post-pandemic EQA for SARS-CoV‑2 virus genome detection was broadly consistent with the previous rounds as most false negative results were reported for the sample with the lowest virus load. When controlling for virus concentration, the results from the two samples with the highest concentration were slightly lower than the expected true positive ratio, but the sample with the lowest virus load was within expectations based on all previous results. When stratified by subsets of results, the observations from earlier rounds that automated test systems had higher detection ratios than manual test systems and that medical laboratories had higher detection ratios than nonmedical laboratories continued in the post-pandemic period. We acknowledge that the design of the post-pandemic schemes varied slightly from those during the pandemic, in a shift towards including other respiratory viruses in the panel. As a result, some participants may have incorporated multiplex tests to detect other respiratory viruses. Although we do not have the statistical power to analyze it here, this could be a potential confounding factor in determining whether performance has decreased relative to previous rounds.

Adding to the analysis presented in a previous study, we now separately analyzed the performance of NPT/POCT assays as a subset of the group of automated test systems. Automated test systems intended for NPT/POCT do not require delicate manual work steps and deliver clear results or a clear indication of a malfunction or measurement error [10]. Therefore, medical professionals without laboratory qualifications are authorized to also use such test systems [15]; however, our results show decreasing detection ratios (true positive results) in the order: automated laboratory systems (98.7%) > automated systems intended for NPT/POCT use (95.0%) > manual methods (89.6%) > in-house assays (73.7%) for samples with relatively low virus load (Table 4). Therefore, the automated systems intended for NPT/POCT use did not meet the expectation to deliver almost perfect performance, were surpassed by automated laboratory systems, but performed better than methods requiring manual steps.

The World Health Organization (WHO) defined a limit of detection (LOD) of NAT test systems of 1000 cp/mL as required and < 100 cp/mL as desirable [11]. In Austria, however, massive testing was prioritized above this recommendation, and the recommended LODs were not declared mandatory. This lack of enforcement of LOD regulations may partly explain why we continued to observe 11% false negative results for samples ~1000 cp/mL in the post-pandemic EQA rounds (Table 3), which is not an improvement over the > 6% false negative results for samples of similar concentration in earlier rounds (Table 4). Given that 25% of symptom-free individuals who were coincidentally identified as positive at screening had low viral loads, using only sufficiently sensitive tests should be required, at least for testing asymptomatic individuals [12–14]. As Austrian laboratories were not incentivized to improve SARS-CoV‑2 diagnostic methods, and the existence of unprecedented shortages of reagents and consumables in the early phases of the pandemic, it is possible that participants could not switch to better performing assays, or were reluctant to do so, even if feedback from participation in the EQAs indicated that their assay of choice had low performance.

However, it must be stated that the EQA schemes we report here were not strictly designed for NPT/POCT assays as they are designed to be implemented on primary human samples. For example, some participants with POCT systems would have had to use a swab to remove some of the fluid from the provided sample, in contrast to methods where RNA could be extracted directly from the provided material and concentrated. Theoretically, this would have diluted the test sample, which may explain the loss of sensitivity for the low-concentration sample for NPT/POCT test systems compared to other automated methods.

We also report the results of nonmedical laboratories and specifically categorize pharmacies as a subset of the group of nonmedical laboratories. As mentioned above, a small fraction of all pharmacies registered to perform SARS-CoV‑2 testing participated in the reported EQA schemes. Of the 359 test results submitted by pharmacies over 6 rounds, 16 (4.5%) were reported from automated test systems, 73 (20.3%) were reported from automated POCT test systems, and the majority (270, 75.2%) were reported from manual test systems, the systems with the lowest overall performance, in general, and those that require the most technical competence; however, when interpreting these findings, it is worth reiterating the fact that we do not know the ultimate motivations of the participants, nor, for example, whether their participation is intended to test/validate new assays not in routine use.

As with all studies on EQA data, a limitation of this study is that results can only be analyzed as they were reported by participants. It must be trusted that they were generated properly. We cannot assume the trends we observed represent the testing performance in Austria, as we do not know if more laboratories than those that participated in an EQA round were in operation and what performance their test systems had. Nonetheless, the data show the dynamics of test performances across laboratory type and assay type from the start of the pandemic. We were limited in our comparisons to previous rounds by statistical sensitivity (or statistical power) due to relatively small sample sizes and small effect sizes. A post hoc power analysis (not shown) suggested that we achieved a power (1 − β) of only 0.29 with a sample size of 327 results comparing whether the observed true positive ratio of 95.4% in the post-pandemic round was significantly different from pandemic rounds (Table 3); however, the principal asset of these data is the existence of > 6000 results available from the beginning of the pandemic. We can say with some confidence that the overall performance is high, and individual laboratories can receive excellent feedback based on this large dataset for monitoring their performance and determining whether improvements are necessary. Our results are similar to those reported by other EQA providers analyzing performance over time for SARS-CoV‑2 nucleic acid testing [17, 18]. Even if we continue to see a decline in response ratios in the upcoming years, our dataset provides essential information for health authorities on the overall quality and accuracy of SARS-CoV‑2 monitoring. This provides confidence for estimating the incidence in the population to monitor trends and dynamics in the virus circulation.

### Supplementary Information

Supplement 1: Numbers of test systems (devices and reagents) used in post-pandemic SARS-CoV‑2 nucleic amplification EQA rounds
