# Supplementary material for: External quality assessments for SARS-CoV-2 genome detection in Austria: A comparison of the first postpandemic round with results from the pandemic era
Source: Wien Klin Wochenschr. 2024 Apr 23;136(15-16):429–38. doi: 10.1007/s00508-024-02353-1 (PMC11327175; doi:10.1007/s00508-024-02353-1)
Supplement: Supplementary file 1 — Supplement 1: Numbers of test systems (devices and reagents) used in post-pandemic SARS-CoV‑2 nucleic amplification EQA rounds [file 508_2024_2353_MOESM1_ESM.docx]

**Supplement 1: Numbers of test systems (devices and reagents) used in post-pandemic SARS-CoV-2 nucleic amplification EQA rounds**

| **Device** | **no.** | **Reagent** | |  |
| --- | --- | --- | --- | --- |
| **Automated laboratory systems (14 participants)** | | | |  |
| Abbott Alinity | 2 | x | Alinity m Resp-4-Plex Assay | |
| BD MAX | 2 | x | BDmax SARS-CoV-2/FLU | |
| Hologic Panther | 1 | x | Aptima SARS-CoV-2/Flu Assay | |
|  | 2 | x | SARS-CoV-2 / Influenza A/B und RSV Assay | |
| NeuMoDX | 3 | x | NeuMoDx Flu A-B/RSV/SARS-CoV-2 | |
|  | 1 | x | NeuMoDx SARS-CoV-2 Test Strip | |
| Roche Cobas 5800 | 1 | x | cobas SARS-CoV-2 Qualitative | |
| Roche Cobas 6800 | 2 | x | cobas SARS-CoV-2 | |
| **Manual methods (22 participants)** | | | |  |
| ABI 7500 | 1 | x | TIB MOLBIOL Light MIX | |
| CFX96 Touch | 1 | x | Allplex 2019-nCoV (Seegene) | |
|  | 1 | x | Real Star R Altona | |
| LightCycler 480 | 1 | x | genesig Real-Time PCR | |
|  | 1 | x | Light Mix Modular | |
|  | 1 | x | QIAprep&amp Viral RNA | |
|  | 2 | x | TIB MOLBIOL Light MIX | |
| LineGene Mini S | 3 | x | PhoenixDx POC SARS-Cov-2 | |
| MIC | 1 | x | Luna Universal Probe One-Step | |
| QuantGene 9600 | 1 | x | Molaccu Covid-19-Detection Kit Zybio | |
|  | 1 | x | Phoenix Dx SARS-CoV-2 Multiplex IVD | |
| QuantStudio 5 | 1 | x | PhoenixDx Cofluenza 4-Plex IVD | |
|  | 1 | x | ViroReal Kit SARS-CoV-2 & SARS | |
| QuantStudio 7 Flex | 1 | x | TaqPath Covid 19 | |
| SLAN - 96P Real-time PCR System | 1 | x | nCov-19 QLP 2.1 RT-PCR Kit | |
|  | 1 | x | Novel Coronavirus (2019n-Cov) NA Diagnostic Kit | |
| STC-96A PLUS | 1 | x | nCov-19 QLP 2.1 RT-PCR Kit | |
| UltraFast QPCR Gerät HC800 (Hecin) | 1 | x | 2019-nCoV-Nukleinsäure-Testkit (Hecin) | |
| Vazyme FMR3 | 1 | x | Vazyme 2019-nCov Triplex RT-qPCR Detection Kit | |
| **Laboratory assays also intended for NPT/POCT use (73 participants)** | | | |  |
| Roche cobas Liat | 6 | x | cobas Liat SARS-CoV-2 und Influenza A/b | |
| Convergys POC RT-PCR | 1 | x | Convergys POC RT-PCR COVID-19/Influenza/RSV Detec | |
| Cepheid GeneXpert | 8 | x | Xpert Xpress CoV-2 plus | |
|  | 14 | x | Xpert Xpress SARS-CoV-2 | |
|  | 5 | x | Xpert Xpress SARS-CoV-2/Flu/RSV | |
|  | 33 | x | Xpert Xpress SARS-CoV-2/Flu/RSV plus | |
| DiaSorin Liaison MDX | 4 | x | SIMPLEXA Covid-19 Direct Reaction Mix | |
| VitaPCR | 1 | x | VitaPCR SARS-CoV-2 Assay | |
|  | 1 | x | VitaPCRInfluenza/SARS-CoV-2 (Flu/SC2) Assay | |
